# HDAC4 is required for inflammation-associated thermal hypersensitivity

**DOI:** 10.1096/fj.14-264440

**Published:** 2015-04-22

**Authors:** Megan Crow, Nikita Khovanov, Jayne H. Kelleher, Simone Sharma, Andrew D. Grant, Yury Bogdanov, John N. Wood, Stephen B. McMahon, Franziska Denk

**Affiliations:** *Wolfson Centre for Age-Related Diseases, King’s College London, London, United Kingdom, ^†^UCL Genomics, ^‡^Molecular Nociception Group, Wolfson Institute for Biomedical Research, University College London, London, United Kingdom

**Keywords:** epigenetics, pain, nerve growth factor, transcription

## Abstract

Transcriptional alterations are characteristic of persistent pain states, but the key regulators remain elusive. HDAC4 is a transcriptional corepressor that has been linked to synaptic plasticity and neuronal excitability, mechanisms that may be involved in peripheral and central sensitization. Using a conditional knockout (cKO) strategy in mice, we sought to determine whether the loss of HDAC4 would have implications for sensory neuron transcription and nociception. HDAC4 was found to be largely unnecessary for transcriptional regulation of naïve sensory neurons but was essential for appropriate transcriptional responses after injury, with *Calca* and *Trpv1* expression consistently down-regulated in HDAC4 cKO compared to levels in the littermate controls (0.2–0.44-fold change, *n* = 4 in 2 separate experiments). This down-regulation corresponded to reduced sensitivity to 100 nM capsaicin *in vitro* (IC_50_ = 230 ± 20 nM, 76 ± 4.4% wild-type capsaicin responders *vs.* 56.9 ± 4.7% HDAC4 cKO responders) and to reduced thermal hypersensitivity in the complete Freund’s adjuvant (CFA) model of inflammatory pain (1.3–1.4-fold improvement over wild-type controls; *n* = 5–12, in 2 separate experiments). These data indicate that HDAC4 is a novel inflammatory pain mediator and may be a good therapeutic target, capable of orchestrating the regulation of multiple downstream effectors.—Crow, M., Khovanov, N., Kelleher, J. H., Sharma, S., Grant, A. D., Bogdanov, Y., Wood, J. N., McMahon, S. B., Denk, F. HDAC4 is required for inflammation-associated thermal hypersensitivity.

Chronic pain is one of the leading causes of disability worldwide (1) and is associated with long-term changes in neuronal excitability (2) and gene expression (3). Epigenetic mechanisms, such as histone posttranslational modification or DNA methylation, may play an important role in mediating these changes, as they are dynamic, instructive for transcription, and potentially long lasting (3, 4). Indeed, there is growing evidence to suggest that drugs targeting chromatin-modifying enzymes can be analgesic in animal models (5–9), particularly histone deacetylase (HDAC) inhibitors. However, it has become increasingly clear that commonly used HDAC inhibitors have very poor selectivity and are capable of targeting only HDAC types 1–3, collectively known as class I (10). Other classes of HDACs, such as classes II and IV, cannot be studied with pharmacological tools, and alternative approaches are needed to delineate their role in pain.

HDAC4 is a class IIa HDAC that functions as a transcriptional corepressor (11–13). In cortical neurons, it regulates the transcription of genes associated with synaptic plasticity (14), and selective knockout of HDAC4 alters neuronal excitability and memory formation (15). The role of HDAC4 in sensory neurons remains largely unexplored. Genetic studies in both the mouse and the nematode have given some indication that HDAC4 and its ortholog *hda4* may be necessary for temperature sensing (16, 17), but the mechanism and cell types responsible for this remain unclear. It is also unknown whether HDAC4 is involved in the emergence of chronic pain. One recent study showed that HDAC4 levels correlate negatively with the severity of osteoarthritis in humans (18), suggesting that it may serve a protective function.

In the current study, we made use of targeted transgenic approaches to investigate whether HDAC4 is essential for sensory neuron function in both naïve and chronic pain states. We crossed 2 separate Cre-driver lines with floxed HDAC4 mice to create a selective knockout in peripheral sensory neurons. One of these lines, the voltage-gated sodium channel Na_v_1.8-Cre line, has been extensively used to target small primarily nociceptive neurons of the dorsal root ganglion (DRG) (19). The other line, Advillin-CreERT2, was developed more recently and allows for inducible panneuronal knockout by administration of the estrogen receptor agonist tamoxifen (20).

Our mouse models therefore enabled us to study the absence of HDAC4 in a spatially and temporally selective fashion. Their phenotypes are reported herein. We found that the absence of HDAC4 attenuates the development of chronic inflammatory pain, on both the transcriptional and behavioral levels.

## MATERIALS AND METHODS

### Animals

All work conformed to United Kingdom Home Office legislation (Scientific Procedures Act 1986) and was performed on animals or tissue taken from 2-mo-old animals, unless otherwise stated in **Table 1**. All lines were backcrossed for 5 generations to pure C57Bl/6J mice, to achieve near congenicity (>95%) before the experiments. For all the experiments, wild-type and knockout groups were age and sex matched.

**TABLE 1.** Age of animals in each study

**Study**	**Strain**	**Age(mo**)
Baseline sensitivity/acute nociception	Na_v_1.8	2–4
Naive		
RNA for microarray	Na_v_1.8	2
Immunohistochemistry	Na_v_1.8	2
CFA - behavior	Na_v_1.8	2
Sciatic nerve crush		
Behavior	Na_v_1.8	2
Immunohistochemistry	Na_v_1.8	6
Baseline sensitivity/acute nociception	Adv	2
CFA		
Behavior	Adv	4–6
ELISA	Adv	>6

### Genotyping

The HDAC4^fl/fl^, Na_v_1.8 Cre, and Advillin-CreERT2 mouse lines have been described, and genotyping was performed according to established protocols (19–21). Genotyping primers are listed in **Table 2**.

**TABLE 2.** Genotyping primers

Primer target	**Forward (5′–3′**)	**Reverse (5′–3′**)
HDAC4^fl/fl^	ATCTGCCCACCAGACTATGTG	CTTGTTGAGAACAAACTCCTGCAGCT
HDAC4 recombined allele	AGGCTGAGGGCAAGTTAGAC	GATTGACCGTAATGGGATAGGTTACG
Na_v_1.8-Cre	TGTAGATGGACTGCAGAGGATGGA	AAATGTTGCTGGATAGTTTTTACTGCC
Wild-type Na_v_1.8	TGTAGATGGACTGCAGAGGATGGA	TTACCCGGTGTGTGCTGTAGAAAG
Advillin CreERT2	CCCTGTTCACTGTGAGTAGG	GCGATCCCTGAACATGTCCATC
Wild-type Advillin	CCCTGTTCACTGTGAGTAGG	AGTATCTGGTAGGTGCTTCCAG

### Tamoxifen dosing

Tamoxifen (T5648; Sigma-Aldrich, Poole, United Kingdom) was prepared according to the protocol of Metzger and Chambon (22). Briefly, 10 mg tamoxifen was dissolved in 100 µl 100% ethanol, made up to 10 mg/ml in autoclaved sunflower oil, and placed on a shaker at room temperature for 2–3 h. The drug was separated into aliquots and stored at −20°C before administration. At 8–20 wk of age, the mice received 2 mg tamoxifen intraperitoneally once daily for 5 d.

### Behavior

All behavioral testing was performed on littermate controls by an experimenter blind to genotype on a per animal (rather than per group) basis. On each day, the animals were randomized into test boxes according to the list randomizer function of Random.org (*http://www.random.org*). Baselines were determined over 3 d. In the case of tamoxifen administration, both knockout animals and their controls received the compound, and testing was performed only after a recovery period of 4 wk.

### Von Frey test

The 50% mechanical thresholds were determined with calibrated Von Frey filaments following the up–down method of Dixon (23) and Chaplan *et al.* (24). The animals were habituated to the testing environment for 60 min before testing. Filaments were applied to the plantar surface of the hind paw for 3 seconds, and a bimodal (yes/no) response was recorded.

### Hargreaves test

Heat withdrawal thresholds were assessed with the Hargreaves apparatus (Ugo Basile, Varese, Italy), set to an infrared intensity of 40 and a cutoff time of 32.5 s. The animals were habituated for at least 30 min before testing, and care was taken to ensure that the glass base was kept clean and that the animals were not in deep sleep when measurements were taken, as sleep has been shown to greatly influence withdrawal latencies (25). At least 3 measurements of withdrawal latency were taken per paw on each test day.

### Tail-flick test

The tail-flick response was measured at 49 or 52°C to determine spinal reflex responses to innocuous warm and noxious heat stimuli, respectively, according to the protocol of Ben-Bassat *et al*. (26). Briefly, mice were restrained, the tail immersed in a water bath, and the latency to respond recorded. Three measurements were recorded on the test day, with at least 5 min between trials.

### Inflammatory pain model

To model chronic inflammatory pain, a 20 µl intraplantar complete Freund’s adjuvant (CFA) (F5881; Sigma-Aldrich) was injected into the right hind paw.

### Sciatic nerve crush model of peripheral nerve regeneration

Under isoflurane anesthesia, the sciatic nerve was exposed and crushed with watchmakers forceps coated in lamp black to mark the crush site. The crush site was kept a constant 37 mm from the tip of the third toe, and the wound was closed with wound clips.

### Pinprick assay

To assess sensory recovery after sciatic nerve crush, the pinprick test was used (27). Under light restraint, 16 areas of the denervated paw were stimulated with a pin, and responses were scored on a 3-point scale (0, no response; 1, light or inconsistent response; and 2, strong consistent withdrawal response).

### DRG dissections

The animals were killed by a fatal overdose of sodium pentobarbital before transcardial perfusion with PBS. A dorsal laminectomy of the spinal cord was performed *in situ*. To identify lumbar L3–L5 DRGs, the sciatic nerve was traced up to the spinal cord, and the 3 ganglia attached were dissected, snap frozen in liquid nitrogen, and stored at −80°C before RNA or protein extraction. For dissociated DRG culture experiments, cultures were washed twice with warm PBS before lysis.

### RNA extraction

RNA extraction was performed in a 2-step protocol consisting of phenol-chloroform extraction followed by cleanup and elution on RNeasy MinElute columns (Qiagen, Manchester, United Kingdom). Care was taken to avoid batch effects by processing samples in matched groups. RNA concentration was measured with a Nanodrop spectrophotometer (ThermoFisher Scientific, Watham, MA, USA), and 500 ng total RNA was used for subsequent first-strand cDNA synthesis reactions with SuperScript III according to manufacturer’s protocol (Life Technologies, Paisley, United Kingdom).

### TaqMan low-density RT-quantitative PCR array cards

Custom TaqMan low-density RT-quantitative (q)PCR array cards were ordered from Life Technologies (probes listed in **Table 3**). On the day of the experiments, cDNA samples were diluted to 2 ng/µl in a final volume of 100 µl PCR mastermix (1×) containing SYBR green in DNAse-free H_2_O (Qiagen). The samples were mixed by pipetting and loaded into wells, and the cards were spun at 1200 rpm for 2 min, sealed, and run on a 7900HT RT-qPCR machine (Applied Biosystems-ThermoFisher Scientific, Foster City, CA). Raw cycle thresholds (CTs) were exported, and relative quantification was performed in Excel (Microsoft, Redmond, WA, USA) by the ∆∆CT method. Multiple experiment viewer software was used to create data visualization tools.

**TABLE 3.** TaqMan probe IDs

Probesets 1–12	Probesets 13–24	Probesets 25–36	Probesets 37–48
Cacna2d1-Mm00486607_m1	P2rx3-Mm00523699_m1	Scn3a-Mm00658167_m1	Tacr1-Mm00436892_m1
Calca-Mm00801462_m1	P2rx4-Mm00501787_m1	Ctss-Mm01255859_m1	Slco1a6-Mm01267368_m1
Ptgs2-Mm00478374_m1	Ngfr-Mm01309635_m1	Vip-Mm00660234_m1	Adcyap1-Mm00437433_m1
Bdnf-Mm04230607_s1	Sgk1-Mm00441380_m1	Reg3b-Mm00440616_g1	Gad2-Mm00484623_m1
Hcn2-Mm00468538_m1	Tac1-Mm01166996_m1	Ccl2-Mm00441242_m1	Gabra5-Mm00621092_m1
Oprm1-Mm00440568_m1	Ntrk1-Mm01219406_m1	Npy-Mm00445771_m1	Gabbr1-Mm00444578_m1
Scn10a-Mm00501467_m1	Ntrk2-Mm00435422_m1	Gfap-Mm01253033_m1	Gapdh-Mm99999915_g1
Scn9a-Mm00450762_s1	Ntrk3-Mm00456222_m1	Sst-Mm00436671_m1	Hprt-Mm00446968_m1
Scn11a-Mm00449377_m1	Trpa1-Mm01227437_m1	Aif1-Mm00479862_g1	Kcns1-Mm00492824_m1
Nos1-Mm00435175_m1	Trpv1-Mm01246302_m1	Pdyn-Mm00457573_m1	Gch1-Mm01322973_m1
18S-Hs99999901_s1	Atf3-Mm00476032_m1	Egfr-Mm00433023_m1	Ngf-Mm00443039_m1
Rest-Mm00803268_m1	Gal-Mm00439056_m1	Ccr2-Mm00438270_m1	Vgf-Mm01204485_s1

### RT-qPCR

RT-qPCR was performed with a LightCycler FastStart DNA MasterPlus SYBR Green I kit (Roche Diagnostics, Burgess Hill, United Kingdom) according to the manufacturer’s protocol on a LightCycler 480 (Roche Diagnostics). All primers used were exon–exon spanning and were tested both for efficiency using a standard curve and for specificity by melting curve analysis and gel electrophoresis of the PCR products (**Table 4**). All primers had an efficiency of 2.0 ± 0.2. The results were determined from plates where no-template controls were used, to ensure that the reagents were contamination free. Triplicate CTs were averaged, and the results were analyzed by the ΔΔCT method (28).

**TABLE 4.** RT-qPCR primers

Target gene	**Forward (5′–3′**)	**Reverse (5′–3′**)
β-2-Microglobulin	GCCTGTATGCTATCCAGAAAACCC	TGTGAGGCGGGTGGAACTGTG
*Hdac4* exon 1–2	TGAACTTAAGGCACTGACGC	AGGATTCAGCAGCTCCACAG
*Hdac4* exon 6–7	CCAGCGATCCCCGCTACTGG	AGGCTGACACCCCACTCTGGG
*Hdac5*	TGTCACCGCCAGATGTTTTG	TGAGCAGAGCCGAGACACAG
*Hdac9*	GCGAGACACAGATGCTCAGAC	TGGGTTTTCCTTCCATTGCT
*Ntrk1*	ATATCTAGCCAGCCTGCACTTTGT	GCTCATGCCAAAGTCTCCA
*Trpv1*	AACCAGGGCAAAGTTCTTCC	CATCATCAACGAGGACCCAG
*Ywhaz*	AGTCGTACAAAGACAGCACGCTAA	AGGCAGACAAAGGTTGGAAGG

### Microarray

RNA was processed by University College London (UCL) Genomics with an Ambion Whole Transcript Expression Kit (Invitrogen-Life Technologies, Carlsbad CA, USA) and hybridized to Mouse Gene 2.0ST Arrays (Affymetrix; Santa Clara, CA, USA) on a GeneChip Fluidics Station 450 (Affymetrix). The chips were read on an Affymetrix GeneChip Scanner. Quality control and analysis were performed with the following bioconductor packages in R: oligo (29) for preprocessing, robust multichip average normalization (30), and various quality controls (including microarray plots, box plots, and principal component analysis) and linear models for microarray data (31) for statistical analysis. Gene Expression Omnibus (GEO) accession: GSE62405 (GEO, National Center for Biotechnology Information, Bethesda, MD, USA).

### Protein extraction and subcellular fractionation

For Western blot analysis, protein was extracted in 0.2% SDS in double-distilled H_2_O with 1× protease inhibitor cocktail (Roche Diagnostics). For cell fractionation studies, DRGs were homogenized in 150 µl lysis buffer A for 5 min [0.5% DTT, protease inhibitors, 10 M (4-(2-hydroxyethyl)-1-piperazineethanesulfonic acid; HEPES), 1.5 mM MgCl_2_, and 10 mM KCl in H_2_O]. The lysates were centrifuged at 1.5 relative centrifugal force (rcf) for 5 min, and the cytoplasmic fraction was removed. The pellets were resuspended in 100 µl of buffer B (buffer A plus 0.5% SDS), sonicated for 2 min, and centrifuged at 3.5 rcf for 5 min, to obtain nuclear-enriched fractions. Protein concentration was assessed by determining absorbance at 280 nm on a Nanodrop spectrophotometer (ThermoFisher Scientific). Protein lysates were stored at −80°C before reduction for 5 min at 100°C in 1× Laemmli buffer, after which they were stored at −20°C.

### Western blot analysis

Protein (20 µg) was run on NuPAGE Novex 10% Bis-Tris Gels (Invitrogen-Life Technologies) and transferred onto 0.45-μm PVDF membranes (Millipore, Stonehouse, United Kingdom). The membranes were blocked in 5% milk and incubated overnight at 4°C with HDAC4 (1:500, sc-11418; Santa Cruz Biotechnology, Dallas, TX, USA), HDAC5 (1:1000, H4538; Sigma-Aldrich,), HDAC9 (1:500, ab18970; Abcam, Cambridge, United Kingdom), α-tubulin (Sigma-Aldrich) or H3 (1:10,000, ab1791; Abcam). After they were washed, the membranes were incubated in secondary antibody for 1 h (1:5000, horseradish peroxidase–conjugated anti-rabbit or anti-mouse; GE Healthcare, Little Chalfont, United Kingdom). The signal was detected using an enhanced chemiluminescence prime kit (GE Healthcare) and visualized with a UVP GelDoc-It Imaging system (Ultra-Violet Products, Cambridge, United Kingdom).

### ELISA

Calcitonin gene-related peptide (CGRP) concentrations were determined with a commercially available kit (589001; Cayman Chemical, Ann Arbor, MI, USA). Briefly, snap-frozen lumbar DRGs were directly homogenized in 200 μl enzyme immunoassay buffer and run in duplicate wells, according to the manufacturer’s instructions.

### Dissociated DRG culture

After cervical dislocation and dorsal laminectomy, the DRGs were dissected into F12 media (Life Technologies-Gibco, Grand Island, NY, USA). Ganglia were digested for 1 h at 37°C in a final concentration of 0.125% collagenase (Sigma-Aldrich) and 0.1 mg/ml DNAse I (Sigma-Aldrich), washed in 3 ml warm F12, and triturated in 2 ml F12 supplemented with 0.3% BSA (Sigma-Aldrich), 1× N2 supplement (Life Technologies-Gibco), and 1× penicillin/streptomycin (Sigma-Aldrich). The cell pellet was resuspended in supplemented F12 with 50 ng/ml mouse nerve growth factor (NGF; R&D Systems, Minneapolis, MN, USA) and plated onto coverslips precoated with poly-l-lysine (Invitrogen-Life Technologies) and 0.01 mg/ml laminin (Sigma-Aldrich).

### Immunocytochemistry

Cultured primary sensory neurons were washed 3 times with warm PBS and fixed with 4% paraformaldehyde (PFA) for 20 min at room temperature. They were then washed in PBS, blocked in 10% normal goat or donkey serum in PBS + 0.2% Triton X+0.1% sodium azide for 30 min and incubated with 1:1000 anti-mouse β-III-tubulin (Promega, Madison, WI, USA) for 1 h, washed and incubated in 1:1000 Alexa-Fluor 488 (Invitrogen-Life Technologies) and 1:10,000 DAPI (Life Technologies) for a further 1 h.

### Calcium imaging

DRG neurons were used for Ca^2+^ imaging experiments after 18–24 h in culture. Cells were incubated in buffer [HBSS with 10 mM glucose and 10 mM HEPES (pH 7.4)] with Fura 2-acetoxymethyl ester (2 μM) and probenecid (1 mM) for 60 minutes at 37°C, and the coverslips were washed and mounted for imaging. Capsaicin was made up to 100 nM in buffer and applied to cells by continuous perfusion. Individual cell fluorescence was measured at 340 and 380 nm excitation and 510 nm emission with a microscope-based imaging system (PTI, Ford, United Kingdom). At the end of each experiment, the cells were challenged with KCl (50 mM) to provide a maximum Ca^2+^ signal against which to normalize responses. Neurons were identified morphologically and were excluded from analysis if they did not respond to KCl.

### Immunohistochemistry

Adult animals were transcardially perfused with freshly prepared 4% PFA in 0.1 M phosphate buffer (PB; pH 7.4-7.7) before dissection. Tissue was postfixed at 4°C for 3 h in 4% PFA in 0.1 M PB, followed by 24 h at 4°C in 20% sucrose solution in 0.1 M PB. Skin samples were blocked in optimal cutting temperature compound (Tissue-Tek; Sakura Finetek, Torrance CA, USA), frozen in liquid nitrogen, and stored at −80°C. Frozen sections were cut at 12 μm on a cryostat. The slides were dried for at least 1 h and stored at −20°C until further processing. The slides were then blocked for 30–60 min in 10% normal goat or donkey serum, incubated overnight at room temperature with anti-rabbit PGP9.5 (1:1000; Ultraclone, Isle of Wight, United Kingdom), incubated for 2 h in anti-rabbit Alexa 488 (1:1000; Invitrogen-Life Technologies), and mounted with Vectashield (Vector Laboratories, Peterborough, United Kingdom).

### Microscopy and image analysis

For each experiment, a minimum of 3 sections from 3 animals per group were used for analysis. To avoid bias, images were taken and processed in a blinded manner: the animals were assigned randomized numbers at the time of dissection that were not revealed until the analysis was completed. Images were taken with a Zeiss Axioplan 2 fluorescence microscope (Zeiss, Jena, Germany). Counting and cell size measurements were performed with Image J (National Institutes of Health, Bethesda, MD, USA) and Axiovision software (Zeiss), respectively.

### Statistical analysis

Statistical analysis was performed with SPSS, version 21 (IBM, Armonk, NY, USA. Single behavioral measures, immunohistochemical counts, and RT-qPCR data were analyzed using 2-sample Student’s *t* test, assuming unequal variances. Tests with repeated measures were analyzed with repeated-measures ANOVA. Data are presented as means ± sem.

## RESULTS

To determine whether HDAC4 is involved in sensory neuron function, we took advantage of 2 strains of HDAC4 cKOs, hereafter referred to as HDAC4^Nav1.8^ and HDAC4^Adv^.

**Figure 1.**
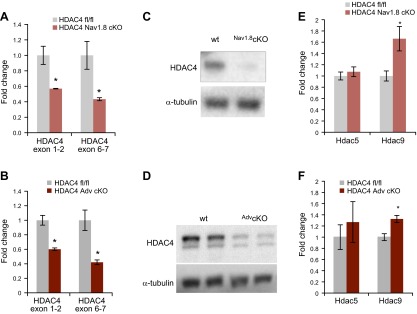
Na_v_1.8- and Advillin-Cre expression successfully causes deletion of *Hdac4^fl/fl^* in the DRGs. *A*, *B*) *Hdac4* mRNA levels were measured by RT-qPCR with exon-spanning primers. Both exon 1 and 2 and the floxed exons 6 and 7 were significantly less expressed in HDAC4 cKOs (*n* = 3–4). *P < 0.05; Student’s *t* test. *C*, *D*) Western blots confirm knockdown of HDAC4 protein in the DRGs. *E*, *F*) The mRNA levels of 2 other class IIa HDACs, *Hdac5* and *Hdac9,* were tested (*Hdac7* is not expressed in the DRGs [32]). *Hdac9* was significantly up-regulated in both strains of HDAC4 cKOs (*n* = 4). wt, wild-type. *P < 0.05; Student’s *t* test.

RT-qPCR and Western blot analysis were used to confirm reduction of HDAC4 expression in HDAC4^fl/fl^ Cre-positive animals (**Fig. 1*A***–***D***). Because of the cell-type specificity of the Cre lines, residual expression of HDAC4 is expected and is likely to arise from nontargeted cells. To determine whether other class II HDACs are up-regulated in the absence of HDAC4, we used RT-qPCR to compare the expression of *Hdac5* and *Hdac9* mRNA between cKOs and wild-type littermate controls. Although *Hdac5* expression was unaffected, there was significant up-regulation of *Hdac9* in both strains of HDAC4 cKOs (Fig. 1*E*, *F*), which may help to buffer the deleterious effects of HDAC4 knockout in these mice. That this could be a possibility was also underscored by subcellular localization data, which indicated that HDAC9 expression is restricted to the nucleus in wild-type DRGs. Meanwhile, HDAC4 and HDAC5 were found in both cytoplasmic and nuclear compartments (Supplemental Fig. 1).

Having established successful knockout of HDAC4, we moved on to investigate its role in normal sensory function. Previous work on HDAC4 has implicated it in regulating the expression of the Runt-related family of transcription factors, which are critical for sensory neuron differentiation (33). For example, forced *Runx1* overexpression in response to the *Tau* promoter leads to a marked reduction of TrkA-positive cells (34). Because Nav1.8-Cre is expressed during embryonic development, we hypothesized that loss of HDAC4 at this time could phenocopy *Runx1*-overexpressing mutants. However, no overt morphologic changes were observed in the DRGs of HDAC4^Nav1.8^ cKOs. (Supplemental Fig. 2).

**Figure 2.**
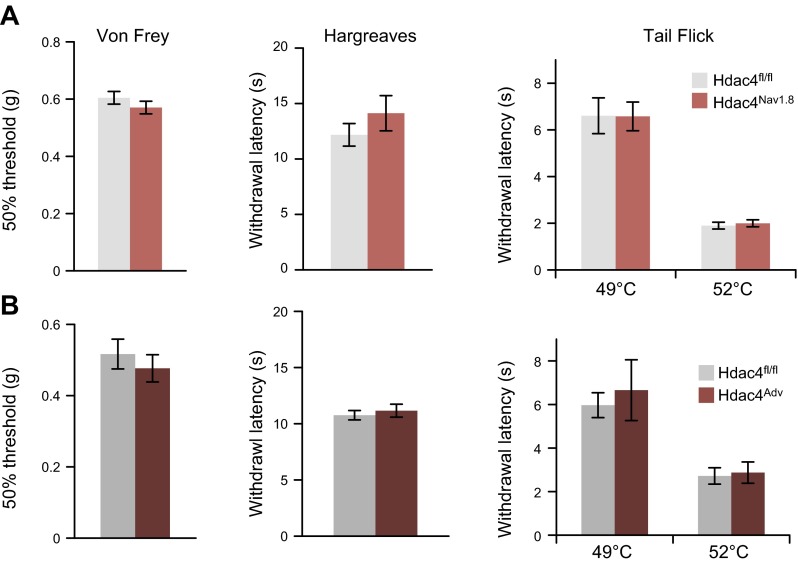
Loss of HDAC4 does not affect baseline somatosensation. Mechanical sensitivity was measured by the Von Frey test; thermal sensitivity was assessed by the Hargreaves and the tail-flick tests. No significant differences were seen between groups across any of these measures for (*A*) HDAC4^Nav1.8^ or (*B*) HDAC4^Adv^ (*n* = 7–30). Nonsignificant, Student’s *t* test.

In addition, previous work has indicated that HDAC4 is necessary for acute sensation of noxious heat, as mice with global knockout of the putative catalytic domain of HDAC4 are insensitive to the hot plate test (16). In our model, however, sensory-neuron–specific knockout of HDAC4 did not alter baseline mechano- or thermosensation, nor did it affect responses to noxious heating of the tail or hind paws (**Fig. 2**). Furthermore, our microarray analysis of lumbar DRGs from naïve adult HDAC4^Nav1.8^ cKOs and wild-type littermates indicated that HDAC4 is not necessary for baseline mRNA expression maintenance in sensory neurons, as no genes were found to be significantly differentially expressed between groups, at a false-discovery rate threshold of *q* < 0.05 (Supplemental Fig. 3). This finding is consistent with that in a previous report on the HDAC4^CamKIIa^ cKO line (15); however, it cannot be separated from the putative compensation by increased levels of HDAC9 mRNA.

**Figure 3.**
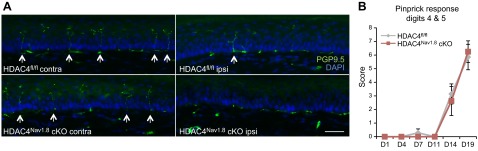
HDAC4 is not necessary for neuronal regeneration *in vivo.* *A*) Six months after sciatic nerve crush, hind paw skin sections were stained with PGP9.5 to mark nerve fibers (green) and DAPI to visualize the epidermal border and assess skin reinnervation. Crossing fibers are indicated by arrows. Scale bar, 50 μm. *B*) Sensory recovery after sciatic nerve crush was measured by the pinprick test. No difference was observed between genotypes.

**Figure 4.**
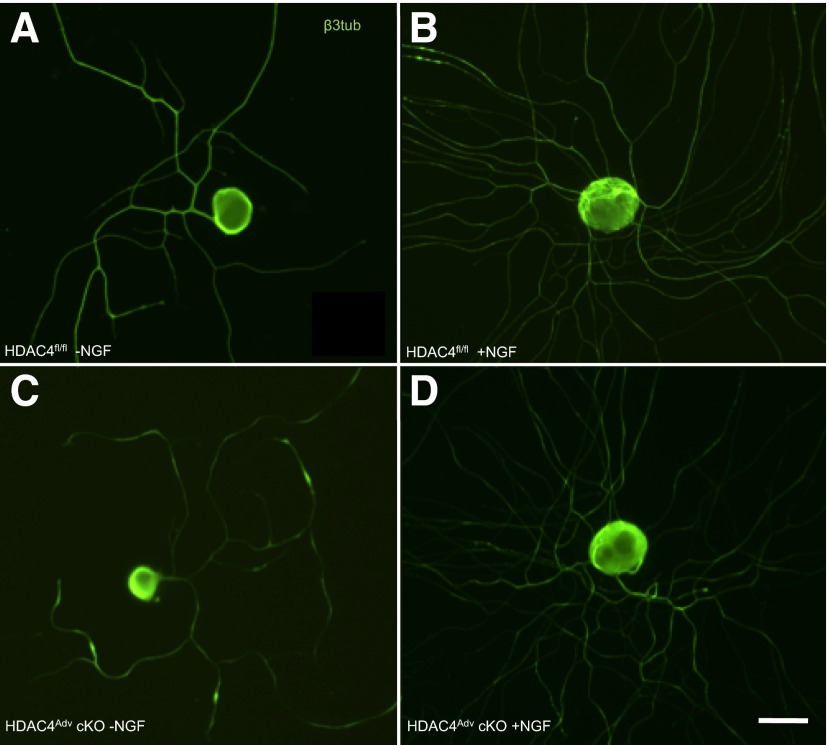
HDAC4 wild-type (*A*, *B*) and cKO (*C*, *D*) neurons were grown in the absence (*A*, *C*) or presence (*B*, *D*) of 100 ng/ml NGF for 24 h, then immunostained for β-III-tubulin. Scale bar, 20 μm.

In summary, our data do not support an important role for HDAC4 in sensory neuron development or normal sensory neuron function. Next, we examined whether it might play a role after injury. Peripheral sensory neurons have the unique capacity to regenerate, and there is strong evidence for the involvement of another class II HDAC, HDAC5, in this process (35, 36). Here, we used the sciatic crush model of nerve injury, which is reversible and causes acute sensorimotor disturbances and denervation of skin afferents (37). Comparison of wild-type and HDAC4^Adv^ cKOs after sciatic crush injury revealed that peripheral expression of HDAC4 is unnecessary for functional recovery, as assessed by the pinprick test and the regrowth of intraepidermal nerve fibers into the hind paw (**Fig. 3**). Consistent with the lack of phenotype *in vivo*, we did not observed any differences in axonal outgrowth between HDAC4^Nav1.8^ cKOs and littermate cultures in an *in vitro* model (**Fig. 4**).

In contrast, we found evidence that HDAC4 may be involved in sensory neuron sensitization. We studied 2 mediators of acute sensitization *in vitro*: NGF and capsaicin. NGF-treated cKO cultures showed differential transcript expression compared to that in the wild-type controls, as measured by custom-designed TaqMan low-density RT-qPCR array cards (Life Technologies). Several genes were affected (**Table 5**), including the high-affinity NGF receptor-1, *Ntrk1*; the gene encoding CGRP, *Calca*; the transient receptor potential vanilloid 1 ion channel,*Trpv1*; and the NGF-inducible peptide, *Vgf.* An independent RT-qPCR for *Ntrk1* confirmed down-regulation of this target (Supplemental Fig. 4*A*). As in the naïve condition, a trend toward up-regulation of *Hdac9* was also observed (Supplemental Fig. 4*B*).

**TABLE 5.** TaqMan array results, arranged by probability

Gene	***P* value**	**Fold change** **(cKO/wt**)
*Ntrk1*	0.00	0.37
*P2rx3*	0.01	0.41
*Gch1*	0.01	0.37
*Trpv1*	0.01	0.41
*Ngf*	0.01	2.04
*Calca*	0.02	0.35
*Kcns1*	0.02	0.36
*Scn9a*	0.02	1.78
*Atf3*	0.02	0.72
*Sst*	0.02	8.04
*Reg3b*	0.03	31.11
*Vip*	0.03	17.24
*Gad2*	0.05	4.76
*Vgf*	0.05	0.14

wt, wild-type.

**Figure 5.**
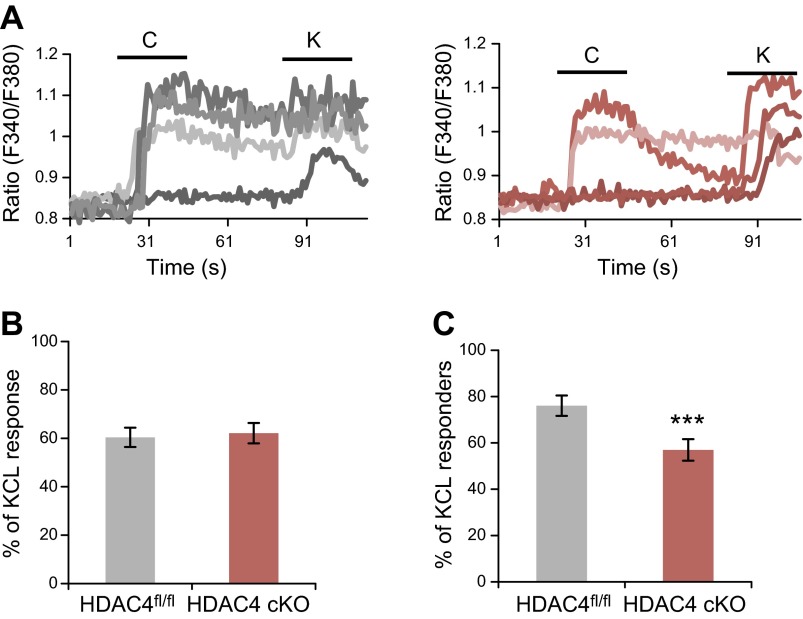
HDAC4 gene transcription and capsaicin sensitivity *in vitro.* *A*) Representative fluorescence traces from calcium imaging experiments. C = 100 nM capsaicin; K = 50 mM KCl. HDAC4^fl/fl^ left, HDAC4^Nav1.8^ cKO right. *B*) Mean peak capsaicin response was plotted as a percentage of the maximum response to KCl. *C*) Cells were considered capsaicin responsive if the capsaicin peak was >20% of the KCl peak. Significantly fewer HDAC4^Nav1.8^ cKO neurons responded to capsaicin treatment (*n* = 5–6). ***P < 0.001; Fisher’s exact test.

TRPV1 is a capsaicin-sensitive cation channel; therefore, its activity in the presence of capsaicin can be monitored using calcium imaging. In concordance with lower levels of *Trpv1* mRNA, significantly fewer HDAC4^Nav1.8^ cKO neurons than wild-type neurons responded to capsaicin treatment *in vitro* (**Fig. 5**). The maximum capsaicin response remained unchanged, suggesting that the reduction in *Trpv1* mRNA expression was more likely to have been caused by the loss of expression in a subset of cells rather than by the reduced expression of *Trpv1* in all capsaicin-sensitive cells.

**Figure 6.**
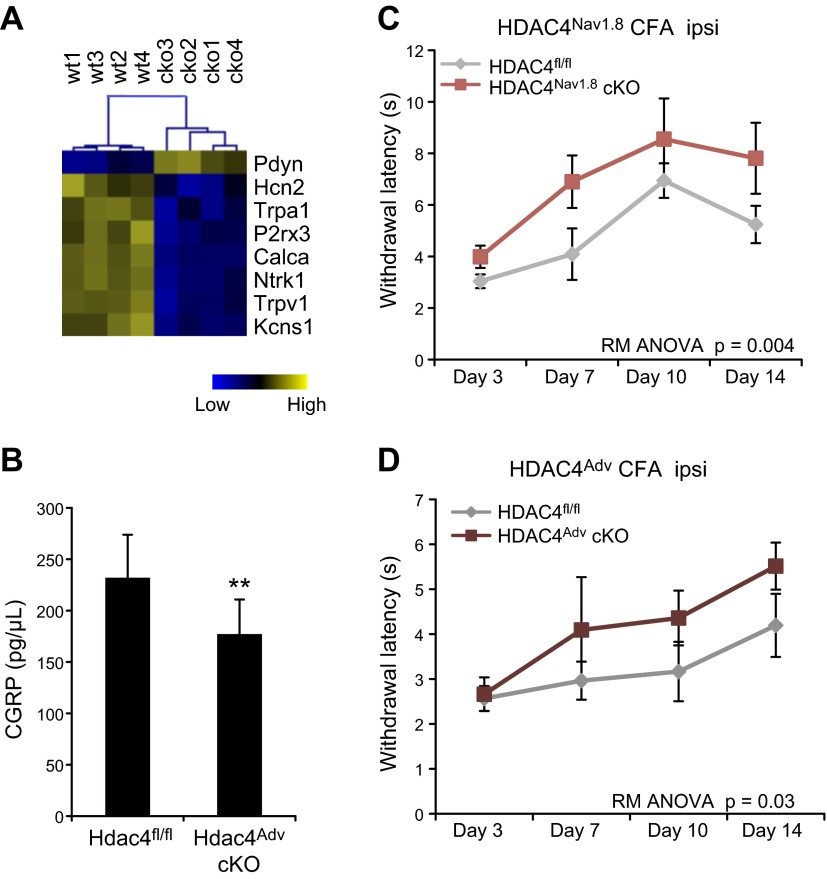
HDAC4 is essential for inflammation-associated transcriptional regulation and thermal hypersensitivity. *A*) Heat map of mean-centered gene expression changes in ipsilateral (ipsi) L3–L5 DRGs at d 15 after CFA. In the absence of HDAC4, many genes were underexpressed compared to their levels in the wild-type controls, including *Calca*, the gene encoding CGRP (*n* = 4). wt, wild-type. *P* < 0.01; Student’s *t* test. *B*) CGRP protein levels were measured by ELISA in ipsi L3–L5 DRGs at d 7 after CFA. The HDAC4^Adv^ cKOs had significantly lower levels of CGRP than did the wild-type controls (*n* = 4/group). **P = 0.006; Student’s *t* test. *C*) Thermal hyperalgesia was measured with the Hargreaves test. Significant attenuation of thermal hypersensitivity was observed in both strains of HDAC4 cKOs compared to that in the wild-type littermate controls: HDAC4^Nav1.8^ (*n* = 5); HDAC4^Adv^ (*n* = 6–12). Repeated-measures ANOVA.

Finally, we used an *in vivo* model of peripheral sensitization, the CFA model. CFA induces chronic inflammation at the site of injection, accompanied by both mechanical and thermal hypersensitivity (38). Results with this model have shown that both TRPV1 and CGRP are crucial for inflammation-evoked thermal hypersensitivity (39). Having observed reduced expression of these transcripts after culturing with NGF, we sought to determine whether HDAC4 is also needed for *Trpv1* and *Calca* mRNA expression *in vivo.* Using TaqMan array cards, we compared mRNA expression of HDAC4^Adv^ cKO and wild-type lumbar DRGs 2 wk after intraplantar injection of CFA. Consistent with *in vitro* results, significantly lower expression of both *Calca* and *Trpv1* was observed in HDAC4^Adv^ cKOs than in similarly affected wild-type littermates (**Fig. 6*A*** and Supplemental Fig. 5). Reduction of CGRP expression was also confirmed at the protein level by ELISA (Fig. 6*B*). A critical finding was that thermal hypersensitivity was significantly attenuated in both strains of HDAC4 cKOs, compared with that in the wild-type littermate controls (Fig. 6*C*, *D*), indicating that HDAC4 is essential for full expression of thermal hypersensitivity, possibly because of its regulatory action on CGRP and TRPV1.

## DISCUSSION

HDAC4 and other class II HDACs have been shown to be involved in several processes that may influence sensory neuron development, pain, and regeneration. We sought to determine the role of peripheral expression of HDAC4 by using a cKO approach in mice.

Our results indicated a clear role for HDAC4 in peripheral sensitization and inflammatory hypersensitivity. In 2 separate strains of HDAC4 cKOs, there was significant transcriptional dysregulation of genes that are involved in pain sensitivity, such as *Calca* and *Trpv1*. Moreover, CFA-induced inflammatory hypersensitivity was attenuated by sensory-neuron–specific HDAC4 knockout.

The precise mechanisms of this process are still unclear. Increased expression of *Calca* and *Trpv1* has been linked to thermal hypersensitivity (36, 37). Down-regulation of these 2 transcripts, as observed in our knockout animals, could therefore be expected to result in the reduced inflammatory responses evinced by our knockout mice. Since HDAC4 negatively regulates transcription, it is unlikely to modulate these targets directly, as deletion would be predicted to result in increased expression. Moreover, a published HDAC4 chromatin immunoprecipitation study in neurons also does not support HDAC4 binding to these genes (40). Instead, the direction of change may imply that HDAC4 is necessary in suppressing another negative transcription factor that in turn reduces *Trpv1* and *Calca* levels. However, a cytoplasmic mechanism cannot be ruled out. It is possible that cytoplasmic HDAC4 promotes sumoylation or deacetylation of TRPV1 or other mediators that regulate its expression. In this case, the loss of HDAC4 could alter the activity of these proteins.

In contrast to peripheral sensitization, there was no evidence of a role of HDAC4 in naïve DRGs. Sensory neuron development, thermo- and mechanosensation, and adult transcriptional profiles were all unaffected by the lack of HDAC4. Neuronal regeneration was also observed to be intact. As noted in the Results, up-regulation of HDAC9 was observed in both knockout mouse strains. Whether HDAC9 provided functional compensation for HDAC4 was not directly addressed in the study—an important caveat for the interpretation of the negative results.

In conclusion, HDAC4 is essential for the full establishment of inflammatory hypersensitivity in mice. The evidence showed that HDAC4 can regulate a variety of important pain mediators, including members of the NGF signaling pathway. Interference with this single upstream regulator could therefore have wide-ranging consequences for pain perception. Further study of HDAC4 and similar epigenetic mediators may open up novel therapeutic avenues.

## Supplementary Material

Supplemental Data

## Abbreviations

*Calca*: calcitonin gene-related peptide gene
CFA: complete Freund’s adjuvant
CGRP: calcitonin gene-related peptide
cKO: conditional knockout
DRG: dorsal root ganglion
HDAC4: histone deacetylase four
L: lumbar (vertebrae)
NGF: nerve growth factor
qPCR: quantitative PCR
PB: phosphate buffer
PFA: paraformaldehyde
rcf: relative centrifugal force
TRPV1: transient receptor potential cation channel subfamily V member 1
*Vgf*: NGF-inducible peptide
