# Supplementary material for: HDAC4 is required for inflammation-associated thermal hypersensitivity
Source: FASEB J. 2015 Apr 22;29(8):3370–8. doi: 10.1096/fj.14-264440 (PMC4511203; doi:10.1096/fj.14-264440)
Supplement: Supplemental Data [file supp_29_8_3370__index.html]

HDAC4 is required for inflammation-associated thermal hypersensitivity — HDAC4 is required for inflammation-associated thermal hypersensitivity — Supplemental Data 

# HDAC4 is required for inflammation-associated thermal hypersensitivity

## Supplemental Data

**Files in this Data Supplement:**

- Supplemental Data
- Supplemental Data
- Supplemental Data
- Supplemental Data
