# Supplementary material for: HDAC4 is required for inflammation-associated thermal hypersensitivity
Source: FASEB J. 2015 Apr 22;29(8):3370–8. doi: 10.1096/fj.14-264440 (PMC4511203; doi:10.1096/fj.14-264440)
Supplement: Supplemental Data [file supp_fj.14-264440_Supplemental_Figure1.pdf]

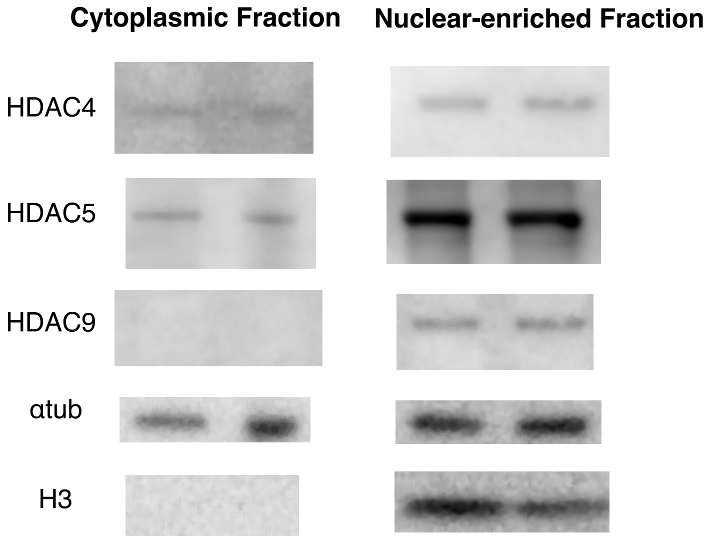

Supplementary Figure 1 – Subcellular localization of class II HDACs in the DRG

A – Representative images from subcellular fractionation Western blots. HDAC4 and HDAC5 could be detected in both protein fractions whereas HDAC9 could only be detected in the nuclear-enriched fraction.
