# Supplementary material for: HDAC4 is required for inflammation-associated thermal hypersensitivity
Source: FASEB J. 2015 Apr 22;29(8):3370–8. doi: 10.1096/fj.14-264440 (PMC4511203; doi:10.1096/fj.14-264440)
Supplement: Supplemental Data [file supp_fj.14-264440_Supplemental_Figure2.pdf]

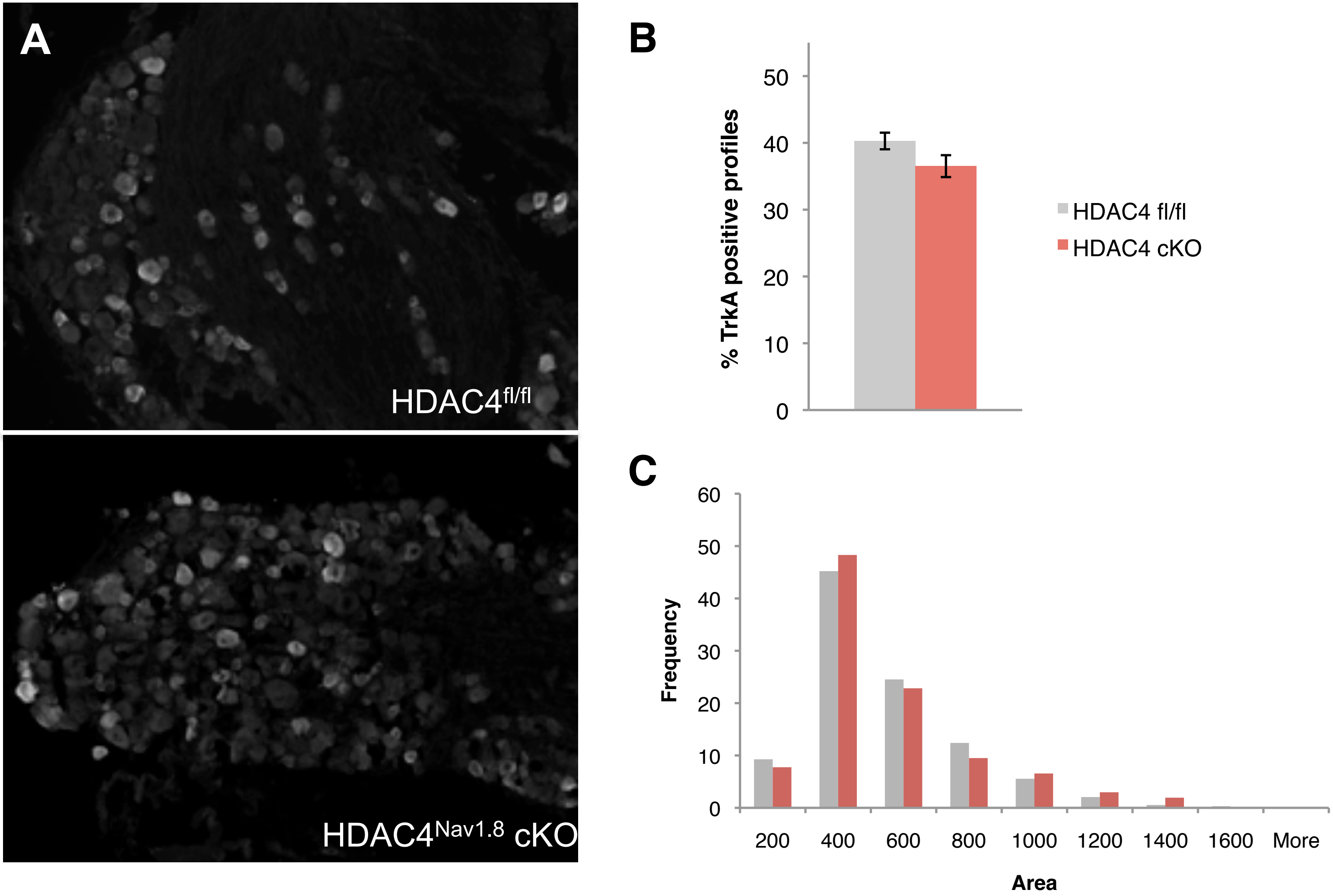

Supplementary Figure 2 - No difference in the proportion of TrkA positive neurons or cell size in $HDAC4^{Nav1.8}$ cKOs compared to littermate controls

A – Whole L4 ganglia from $HDAC4^{Nav1.8}$ cKO and $HDAC4^{fl/fl}$ animals were stained for TrkA. Scale bar represents 100 μM. **B** – Quantification of immunofluorescence. Similar proportions of DRG cell profiles were found to be TrkA positive (40.3%, 36.5%). **C** – Similar cell size distributions were observed between the two genotypes
