# Supplementary material for: HDAC4 is required for inflammation-associated thermal hypersensitivity
Source: FASEB J. 2015 Apr 22;29(8):3370–8. doi: 10.1096/fj.14-264440 (PMC4511203; doi:10.1096/fj.14-264440)
Supplement: Supplemental Data [file supp_fj.14-264440_Supplemental_Figure3.pdf]

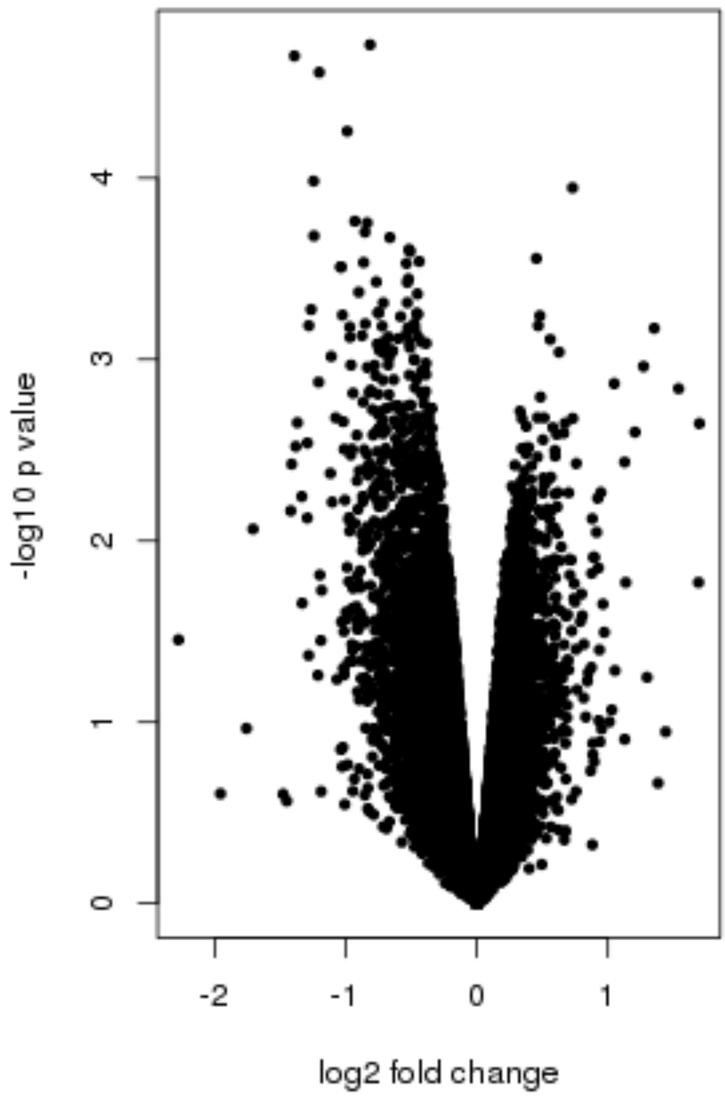

Supplementary Figure 3 – HDAC4 knockout does not incur mass transcriptional alterations in naïve adult DRG
Volcano plot of microarray results. A microarray was performed to seek global transcriptional differences between adult $HDAC4^{Nav1.8}$ cKO DRGs and wildtype DRGs (n=3/group). No genes passed multiple hypothesis test correction.
