# Supplementary material for: HDAC4 is required for inflammation-associated thermal hypersensitivity
Source: FASEB J. 2015 Apr 22;29(8):3370–8. doi: 10.1096/fj.14-264440 (PMC4511203; doi:10.1096/fj.14-264440)
Supplement: Supplemental Data [file supp_fj.14-264440_Supplemental_Figure4.pdf]

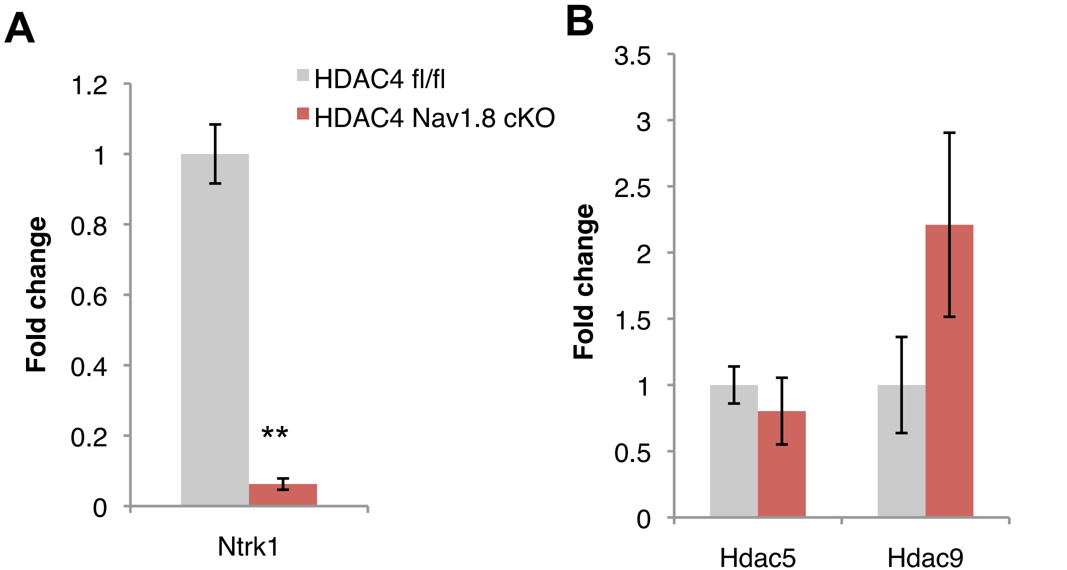

Supplementary Figure 4 – Class II HDAC mRNA expression in cultured neurons

RT-qPCR for *Ntrk1* and class II HDAC mRNA levels (see Table 5*)*. ***A*** – Significant downregulation of Ntrk1 was observed in an independent RT-qPCR, validating this result from the Taqman array. **B** – Neither class II HDAC showed significantly different expression between groups, although there was a trend for upregulation of Hdac9 as observed in vivo (n=6-8, n.s., Student's t-test).

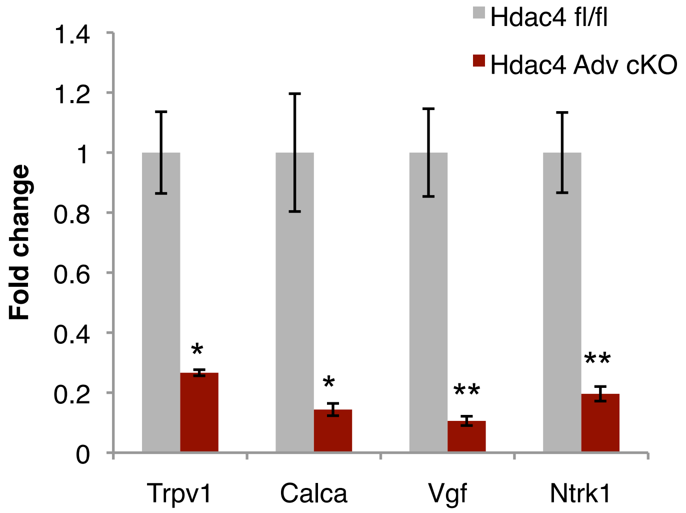

Supplementary Figure 5 – TaqMan array results replicate in an independent RT-qPCR experiment

RT-qPCR results from L3-L5 ipsilateral DRGs at day15 after CFA (n=4). Significant downregulation of Trpv1 (p=0.01), Calca (p=0.02), Ntrk1 (p=0.008), Vgf (p=0.008) was observed in HDAC4 cKOs compared to wildtype controls (Student's t-tests), confirming Taqman array card results (Fig 6)
